# Bilaterale seröse Netzhautablösung: mögliche Folge einer Parvovirus-Infektion?

**DOI:** 10.1007/s00347-020-01314-z

**Published:** 2021-01-14

**Authors:** V. Schöneberger, A. Händel, T. Osterholt, P. Brinkkötter, L. Altay

**Affiliations:** 1 Zentrum für Augenheilkunde, Medizinische Fakultät und Uniklinik Köln, Universität zu Köln, Köln, Deutschland; 2 Klinik II für Innere Medizin, Medizinische Fakultät und Uniklinik Köln, Universität zu Köln, Köln, Deutschland

## Falldarstellung

### Anamnese und klinischer Erstbefund

Die ophthalmologische Erstvorstellung des 37-jährigen männlichen Patienten erfolgte konsiliarisch bei hypertensiver Entgleisung unklarer Genese am Bett. Aufgrund seines deliranten Zustandes konnte keine Anamnese erhoben werden. Die Sehschärfe betrug beidseits (bds.) lediglich Handbewegung. Es zeigten sich klare brechende Medien und ein reizfreier Vorderabschnitt. Fundoskopisch imponierte an beiden Augen eine zirkulär verstrichene Papille mit Punkt- und Fleckblutungen sowie Exsudaten in allen Quadranten und Gefäßtortuositas, der Fundus wirkte ödematös, und es zeigte sich eine seröse Amotio. Es waren keine Foramen, Zellen oder Infiltrate im Glaskörper zu sehen. Nach Rücksprache mit den internistischen Kollegen erfolgte zur weiteren Abklärung eine infektiologische Diagnostik.

Im weiteren Verlauf gab der Patient an, ursprünglich aus Nigeria zu stammen. Während eines 2‑wöchigen Aufenthaltes in seiner früheren Heimat entwickelte er ein starkes Krankheitsgefühl mit grippeähnlichen Symptomen sowie Sehstörungen in Form von Doppelbildern. Die ophthalmologische Vorgeschichte war bis zu diesem Zeitpunkt leer.

Weitere internistische und infektiologische Diagnostik ergab den Nachweis einer Parvovirus-Erstinfektion. Diese führte bei dem Patienten zu einer Enzephalitis mit passagerem Delir sowie zu einer thrombotischen Mikroangiopathie (TMA) mit Hämolyse und Thrombozytopenie. Eine daraus resultierende maligne Hypertonie führte zur Nierenschädigung, deren Funktion mittels passagerer intermittierender Hämodialyse überbrückt werden musste (Tab. 1).

**Table 1:** 

Diagnosen	Befunde	
Parvovirus-B19-Infektion	*Klinik:*	Fieber, Abgeschlagenheit, Appetitlosigkeit, Delir, Enzephalitis, bilaterale seröse Netzhautablösung
	*Serologie:*	Nachweis von > 100 Mio. i.U./ml
		IgM positiv, IgG negativ
		Nachweis von Parvovirus B19 im Liquor
	*Enzephalitis mit Delir*	cMRT: a. e. embolisch subakute Ischämien bihemisphärisch supratentoriell und rechts zerebellär mit FLAIR-Demarkation. Diffuse FLAIR-hyperintense Signalanhebungen im Hirnstamm, möglicherweise im Rahmen einer Hirnstammenzephalitis (DD Bickerstaff-Enzephalitis)
	Kein Hinweis auf eine aplastische Anämie	
Thrombotische Mikroangiopathie(TMA)-Konstellation persistierend trotz adäquater Blutdrucksenkung	Laborchemisch: Thrombozyten 78 Tsd/µl, Hämoglobin 6,7 g/dl, Retikulozyten normwertig, Fragmentozyten 14 ‰, supprimiertes Haptoglobin, Kreatinin 6,34 mg/dl, LDH 612 U/l	
	Ausschluss eines hämolytisch-urämischen Syndroms durch Shiga-Toxin produzierende *E.-coli*-Stämme (STEC-HUS)	
	Ausschluss einer thrombotisch-thrombozytopenischen Purpura (ADAMTS-13-Aktivität = 62 %)	
	Coombs-Test negativ, Malaria negativ	
	Ausschluss Hämoglobinopathien	
Maligne Hypertonie	Normwertiger Renin/Aldosteron-Quotient, normwertiges Kortisol	
	Ausschluss Nierenarterienstenose	
	A. e. im Rahmen einer thrombotischen Mikroangiopathie	
Akute Nierenschädigung	KDIGO Stadium 3 mit intermittierender Dialysepflichtigkeit	
Schwere Hyponatriämie	119 mmol/l	

### Diagnose

Es lag eine beidseitige seröse Netzhautablösung vor, die im Rahmen der Parvovirus-Erstinfektion ausgelöst wurde. Differenzialdiagnostisch wurden eine rhegmatogene Genese sowie eine CMV-Infektion ausgeschlossen.

### Verlauf

**Figure 1:**
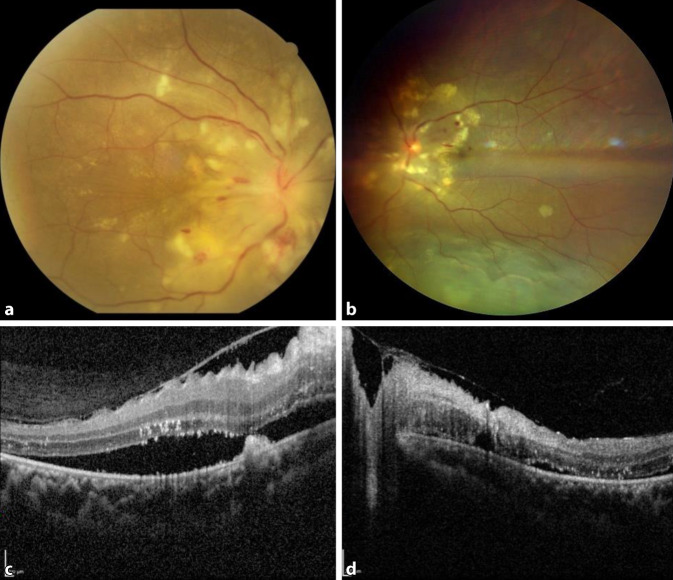

Mit abnehmender Viruslast verschwanden die Symptome des Delirs sowie die Hämolysekonstellation. Beschwerdeführend war dann die persistierende Visusminderung. Zwei Wochen nach Erstuntersuchung besserte sich der Visus bds. auf 1/35 Metervisus. Fundoskopisch zeigten sich bds. eine symmetrische seröse Amotio bis knapp zum unteren Gefäßbogen sowie massive Exsudationen, Cotton-Wool-Herde und Fleckblutungen (Abb. 1a, b). Am rechten Auge waren eine traktive Membran im papillomakulären Bündel und ein Papillenödem zu sehen. In der optischen Kohärenztomographie zeigte sich rechts eine pathologische Glaskörpergrenzmembran mit Netzhautfalte und subretinaler Flüssigkeit (SRF) (Abb. 1c). Am linken Auge war der Befund ruhiger mit geringer SRF am hinteren Pol (Abb. 1d).

**Figure 2:**
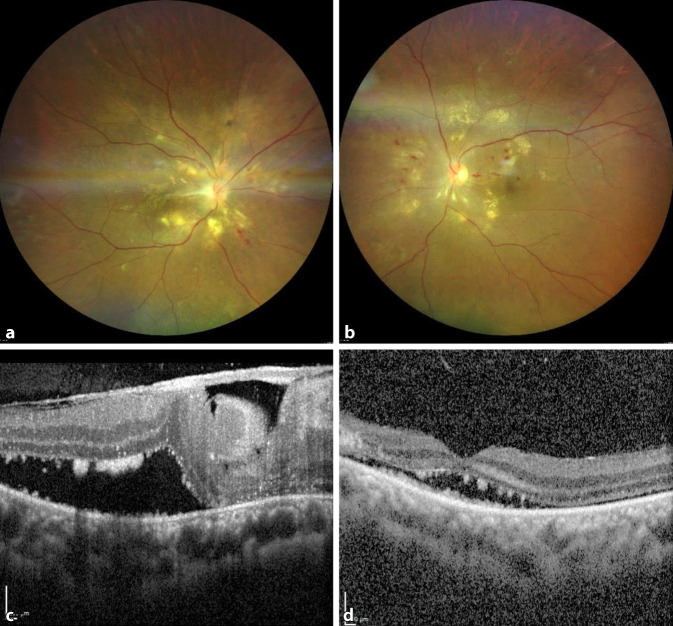

Wir entschieden uns, im Rahmen weiterer Verlaufskontrollen auf eine Spontanresorption der serösen Netzhautabhebung zu warten. Vier Wochen später waren die seröse Amotio und die SRF rückläufig (Abb. 2), und der Visus besserte sich auf 1/25 Metervisus. Die durchgeführte Fluoreszeinangiographie zeigte eine verzögerte Füllung der retinalen Arterien, eine deutliche Hyperfluoreszenz der Papille mit Papillenleckage (R > L) sowie eine gestörte Blut-Retina-Schranke.

**Figure 3:**
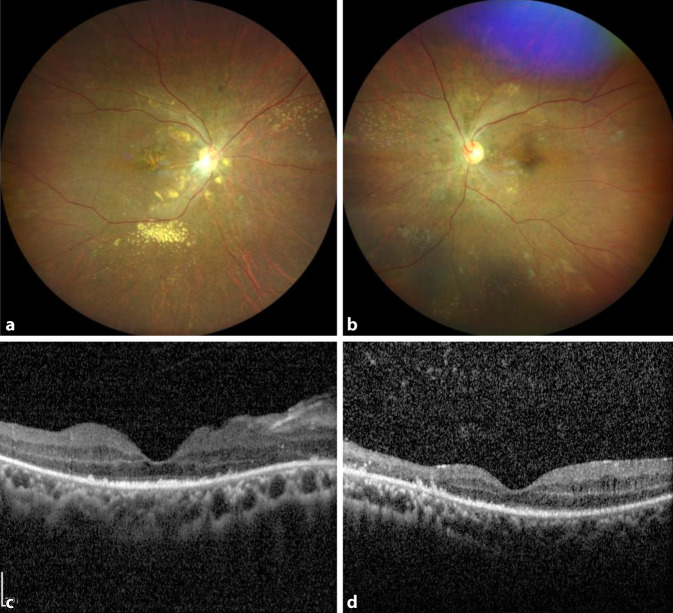

In 3 weiteren Verlaufskontrollen (jeweils mit ca. 4 bis 6 Wochen Abstand) zeigte sich die seröse Amotio mitsamt Exsudationen abnehmend. Der Visus stieg auf 0,2 rechts- und 0,32 linksseitig. Auch die traktive Membran im papillomakulären Bündel des rechten Auges als Komplikation der massiven serösen Exsudationen und der pathologischen Glaskörperabhebung zeigte sich rückläufig (Abb. 3). Wir empfahlen daher, keine operativen Maßnahmen durchzuführen, und planten die nächste Verlaufskontrolle in 6 Monaten.

## Diskussion

Eine Infektion mit Parvovirus B19 verläuft normalerweise asymptomatisch oder mit leichten grippeähnlichen Symptomen. Als häufigste Manifestation der Infektion zeigt sich bei Kindern das Erythema infectiosum, auch bekannt als Ringelröteln, und zählt damit zu den klassischen pädiatrischen Exanthemerkrankungen [1]. Nach durchgestandener Infektion besteht eine lebenslange Immunität, die Seropositivität liegt in Deutschland bei ca. 70 % [2]. Eine Impfung ist zurzeit nicht verfügbar. Die Diagnostik erfolgt üblicherweise klinisch. In unserem Fall wurde eine serologische Sicherung mittels EDTA-Blut mit Parvovirus-DNA mit mehr als 1 × 10^8^ IU/ml durchgeführt. Hinweise auf eine andere Grunderkrankung als Ursache der TMA-Konstellation wurden nicht gefunden. Insbesondere zeigte sich kein Hinweis auf das Vorliegen einer aplastischen Anämie, welche im Rahmen von Parvovirus-B19-Infektionen geschildert wird [1]. Nur selten ist ein solch komplikativer Verlauf zu sehen.

In der Literatur konnte lediglich ein weiterer Fall mit Assoziation einer akuten Parvovirus-B19-Infektion und dem Auftreten einer serösen Netzhautablösung gefunden werden, wobei hier der Befund einseitig, temporal superior gelegen und ohne retinale Exsudate war. Die 28-jährige Patientin wurde aufgrund einer zunehmenden serösen Amotio mittels Vitrektomie und SF6-Gastamponade erfolgreich versorgt. Es wurden dabei Parvovirus-IgM-Antikörper und virale DNA innerhalb der Glaskörperflüssigkeit gefunden [3].

Bei einer beidseitigen serösen Netzhautabhebung ist es obligatorisch, systemische und okuläre Erkrankungen differenzialdiagnostisch auszuschließen. Zu nennen ist hierbei v. a. das Vogt-Koyanagi-Harada-Syndrom, bei dem eine T‑Zell-vermittelte Autoimmunantwort ebenfalls eine Papillitis, neurologische Symptome (z. B. Meningitis) und Hautbefunde (u. a. Vitiligo, Alopezie) auslösen kann [4]. Eine posteriore Skleritis ist nur in 50 % bds. vorzufinden und mit ausstrahlenden, bohrenden Schmerzen verbunden und zeigt als Charakteristikum das „T-Zeichen“ in der sonographischen Untersuchung [5]. Die akute posteriore multifokale plakoide Pigmentepitheliopathie (APMPPE) zeigt fundoskopisch initial multiple, unscharf begrenzte, cremefarbene Läsionen, ein Papillenödem kann vorgefunden werden. In der Fluoreszenzangiographie sind in der Frühphase Hypofluoreszenzen, später Hyperfluoreszenzen im Bereich der Läsionen zu sehen [6]. Das uveale Effusionssyndrom würde sich differenzialdiagnostisch typischerweise mit diffusen Pigmentationen („Leoparden-Spots“) zeigen [7].

Nach Ausschluss der oben genannten Differenzialdiagnosen einer beidseitigen serösen Netzhautablösung und der diagnostizierten Primärinfektion mit Parvovirus B19 ist schlussendlich zu diskutieren, ob der Befund direkt im Bulbus durch die Parvoviren ausgelöst wurde oder der allgemein komplikative Verlauf mit hypertensiver Krise und thrombotischer Mikroangiopathie den ophthalmologischen Befund bedingte. Da wir, anders zum zitierten Case Report [3], keine operative Therapie durchführten, gibt es auch keinen intraokulären Nachweis der Parvoviren. Aufgrund der Enzephalitis wurde eine Lumbalpunktion durchgeführt, die einen Parvovirus-B19-positiven Befund erbrachte. Ein intraokuläres Vorliegen der Viren ist somit denkbar. Eine Alternativhypothese ist, dass die durch die Infektion ausgelöste hypertensive Krise zu der serösen Netzhautablösung führte. Durch Vasokonstriktion und fibrinoide Nekrosen der choroidalen Gefäße kommt es zur Ischämie des darüber liegenden RPE (Elsching-Spots) und dadurch zu einer Leckage in den subretinalen Raum [8]. Ebenfalls ist eine Form der Angiopathia retinae traumatica Purtscher in Betracht zu ziehen. Typisch ist ein plötzlicher Visusverlust, der meist 1 bis 2 Tage nach einem Trauma mit herabgesetzter Mikrozirkulation auftritt, wie es bei unserem Patienten aufgrund der entwickelten thrombotischen Mikroangiopathie der Fall war. Fundoskopisch werden auch Cotton-Wool-Herde, Netzhautblutungen und sog. Purtscher-Flecken (weißliche Exsudate) und ggf. ein Papillenödem gesehen. Nicht beschrieben wird eine seröse Netzhautablösung [9]. Denkbar ist auch eine Kombination beider pathophysiologischer Vorgänge. Ob die beidseitige seröse Netzhautablösung mit Exsudationen, Cotton-Wool-Herden und Fleckblutungen mit massivem Visusverlust am Ende durch die Parvovirus-B19-Erstinfektion direkt ausgelöst wurde oder durch ihren komplikativen Verlauf, bleibt offen. Ein abwartendes Verhalten unter engmaschigem interdisziplinärem Monitoring ist aus unserer Sicht in beiden Fällen zu empfehlen.

## Fazit

- Bei einer beidseitigen serösen Netzhautablösung sollte eine infektiologische Diagnostik erfolgen.
- Das Vogt-Koyanagi-Harada-Syndrom, eine posteriore Skleritis, die akute posteriore multifokale plakoide Pigmentepitheliopathie (APMPPE) sowie das uveale Effusionssyndrom sind bei einer beidseitig auftretenden serösen Netzhautablösung differenzialdiagnostisch in Erwägung zu ziehen.
- Bei einer serösen Amotio im Rahmen einer Parvovirus-Erstinfektion ist zunächst ein abwartendes Verhalten zu empfehlen.
